# Supplementary material for: An Inventory of South African Medicinal Plants Used in the Management of Sexually Transmitted and Related Opportunistic Infections: An Appraisal and Some Scientific Evidence (1990–2020)
Source: Plants (Basel). 2022 Nov 25;11(23):3241. doi: 10.3390/plants11233241 (PMC9738887; doi:10.3390/plants11233241)
Supplement: Supplementary file 1 [file plants-11-03241-s001.zip › plants-1991267-supplementary.pdf]

Supplementary File: Table S1: South African medicinal plants used in the treatment and management of sexually transmitted infections and related opportunistic infections

Family	Medicinal plants	Growth form	Plant part used, and Vernacular names	Mode of preparation	Main, related use(s) and references	Number of mentions
**Acanthaceae**	*Blepharis diversispina* (Nees) C.B.Clarke	H	Roots; Mookapitsi (S)	Boiled and taken orally	Various unidentified STIs, chlamydia and "makgoma" [19, 59].	2
	Blepharis subvolubilis C.B.Clarke	H	Whole plant; Eyelash Flower (E)	Not mentioned	Various unidentified STIs [87]	1
**Amaranthaceae**	*Alternanthera pungens* Kunth	H	Roots; Motsweetswee (S); Tshehlo (S); Burweed (E); Dubbeltjie (A); Ikhungele (Z); Kahkikweek (A); Kakiedubbeltjie (A)	Boiled and taken orally	Gonorrhoea [45, 78, 79, 83, 65].	5
	Amaranthus spinosus L.	H	Fresh leaves; Thepe (S); Sere Pelele (S); Spiny amaranth (E); Needle Burr (E); Soldier Weed (E)	Immersed in water and drunk	General opportunistic infections associated with HIV-AIDS [57, 58].	2
	Gomphrena celosioides Mart.	H	Leaves; Lepolomo la naga (S); Makunye (S); Globe Amaranth (E); Intandangulube (Z); Kakie-onkruid (A); Knopamarant (A)	Boiled and taken orally	Various unidentified STIs [41].	1
**Amaryllidaceae**	*Ammocharis coranica* (Ker Gawl.) Herb.	H	Roots; Icukudo (Z); Incukudwane (Z); Umbhodiya (Z); Boka (S);	Boiled and taken orally	Impotence [46, 81].	2

			Berg Lily (E); Ground Lily (E); Sore-eye flower (E);			
	Boophane disticha (L.f.) Herb.	H	Whole plant; century plant (E); Gifbol (A); Motlatsisa (S); Incumbe (Z); incotho, Incwadi (X, Z)	Boiled and taken orally	Hepatitis B [73].	1
	Clivia miniata (Lindl.) Bosse	H	Bulbs; Boslelie (A); Clivia (E); Fire Lily (E); September Lily (E); Ubuhlungu-beyimba (Z); Umayime (Z)	Not mentioned	Opportunistic infections associated with HIV-AIDS [75].	1
	Cyrtanthus obliquus (L.f.) Aiton	H	Bulbs; Umathunga (X); Knysna lily (E); Knysnalelie (A); Umathaga (Z)	Boiled and taken orally.	Various unidentified STIs [39, 40].	2
	Gethyllis namaquensis (Schönland) Oberm.	H	Bulbs; Nak tsa tholo (S)	Not mentioned	Gonorrhoea [80, 84].	2
	Tulbaghia acutiloba Ha rv.	H	Whole plant; Itsweli (Z); Isivimbampunzi (Z); Ishalati lezinyoka (Z); Isihaqa(Z)	Not mentioned	Impotence [61].	1
**Anacardiaceae**	*Harpephyllum caffrum Bernh.*	T	Stem bark; Ungwenya (Z); Sour plum (E); Suurbessie (A); Suurpruim (A); Umgwenya (X)	Boiled and taken orally	Gonorrhoea [39,40].	2
	Lannea discolor (Sond.) Engl.	T	Roots; Bakhout (A); Isiganganyane (Z); Live-long (E); Mokgôkgôthwane (S); Munii (Tsh),	Root is infused and drunk	Impotence for both men and women [53].	1

	Lannea schweinfurthii (Engl.) Engl. var. *stuhlmannii* (Engl.) Kokwaro	T	Root bark; Bakhout (A); Bastard Marula (E); False Marula (E); Mmopu (S); Mulivhadza (TSH); Tree Grape (E); Umganunkomo (Z); Bolebatsa (S)	Boiled and taken orally	Various unidentified STIs [49].	1
	Ozoroa paniculosa (Sond.) R.Fern. & A.Fern.	T	Roots; Monoko (S); Resin tree (E); Gewone Harpuisboom (A); Isifice (Z); Isifuku (X); Monyêladiatla (S); Mubandulakhali (Tsh)	Boiled and taken orally	Various unidentified STIs [41].	1
	Mangifera indica L.	T	Roots; Momango (S); Mango (E)	Not mentioned	A root decoction is drunk to treat various unidentified STIs [53].	1
	Ozoroa engleri R. Fern. & A. Fern.	T	Roots; Mudumbula (Tsh); Isifice (Z); Drooping Resin Tree (E); Treur-harpuisboom (A)	Ground and boiled in water, used as mouth wash	Opportunistic infections associated with HIV-AIDS [44, 57].	2
	Ozoroa paniculosa (Sond.) R.Fern. & A.Fern.	T	Roots; Bushveld ozoroa (E); Isifice (Z); Monoko (S); Mudumbula (Tsh); Common Resin Tree (E); Monyêladiatla (S); Mubandulakhali (Tsh)	Not mentioned	Various unidentified STIs and impotence [65].	1
	Ozoroa sphaerocarpa R. Fern. & A. Fern.	T	Stem bark; Monoko (S); Currant Resin Tree (E); Isifice (Z); Resin Tree (E); Tar Berry (E)	Pounded and taken orally	Impotence [81].	1

	Sclerocarrya birrea (A.Rich.) Hochst. subsp. *caffra* (Sond.) Kokwaro	T	Stem bark; Morula (S); Umganu (Z); Cider tree (E); Marula (E), Moroelaboom (A), Maeroola (A); Mufula (Tsh)	Boiled and taken orally	Various unidentified STIs, gonorrhoea, impotence and as an immune booster for patients with HIV-AIDS [19, 46, 65, 80, 82, 83, 85].	7
	Searsia lancea (L.f.) F.A.Barkley	T	Roots; Mokalabata (S); Karee (E); Karee (A)	Not mentioned	"*Nta-shete*" [80, 83].	2
**Annonaceae**	*Annona senegalensis* Pers.	SH	Roots; Muembe (Tsh)	Boiled and taken orally until infections subsides	Impotence, syphilis, and chlamydia [50, 54, 75, 85, 95].	5
	Artabotrys brachypetalus Benth.	T	Roots; Mudzidzi, Munna-mutswu (Tsh); Monnamoso (S)	Boiled and taken orally, may also be licked	Impotence for men [50].	1
	Hexalobus monopetalus (A.Rich.) Engl. & Diels	SH	Roots; Baboon's Breakfast (E); Bastersuikerappel (A); Mowedika (S); Muhuhuma (Tsh); Shakama Plum (E)	Boiled and taken orally	Impotence in men [49, 53].	2
**Apiaceae**	*Arctopus echinatus* L.	H	Roots; Bear's Foot (E); Octopus (E); Poxthorn (E); Bitterwortel (A); Platannadoring (A)	Boiled and taken orally	Various unidentified STIs [90].	1
	Arctopus monacanthus Carmich. ex Sond.	H	Roots; Platannadoring (A); Bear's Foot (E)	Boiled and taken orally	Syphilis and gonorrhoea [30, 93].	2
	Centella asiatica (L.) Urb.	H	Whole plant; Bolila-balinku (S); Ibdiab	Not mentioned	Various unidentified STIs, syphilis [62, 90, 94].	3

			Pennywort (E); Marsh Pennywort (E); Icudwane (Z), Varkoortjies (A), Waternael (A)			
	Foeniculum vulgare Mill. var. *vulgare*	H	Leaves; Bobbejaanvinkel (A); Fennel (E); Imbozisa (Z); Vinkel (A); Vinkelbossie (A)	Boiled as a used to wash infected body parts	Oral and genital thrush associated with HIV-AIDS [9].	1
	Heteromorpha aborescens (Spreng.) Cham. & Schltdl.	T	Roots and leaves; Mohlatlabanna (S); Muthathavhanna (Tsh)	Boiled and taken orally, may also be licked	Impotence in men [50].	1
**Apocynaceae**	*Asclepias fruticosa* L.	SH	Roots; Tontelbos (A); melkbos (A); ulusinga lwesalukazi (Z); lebeyana (S); modimolo (S)	Roots are boiled and taken orally	Impotence in men [46].	1
	Carissa bispinosa (L.) Desf. ex Brenan	SH	Roots; Mothokolo o monnyane (S); Forest num-num (E); Bosnoemnoem (A); Isibethankunzi (Z); Isabetha (Z)	Decoction taken orally	Chlamydia [59].	1
	Carissa edulis (Forssk.) Vahl	SH	Roots and leaves; Mothokolo (S); Small num-num, (E); Umlugulu (ND); Murungulu (Tsh)	Decoction taken orally	Various unidentified STIs and body sores associated with HIV-AIDS [19].	1
	Catharanthus roseus (L.) G.Don	H	Roots; Lepolomo le le pinki la drop (S); Liluvha (Tsh); Muluvha (Tsh); Madagascar periwinkle	Decoction taken orally	Various unidentified STIs, syphilis, gonorrhoea, chlamydia, genital warts, dropsy and opportunistic	**19**

			(E); Rosy periwinkle (E); Cape periwinkle (E)		infections associated with HIV-AIDS [19, 38, 45, 49-53, 56, 60, 62, 64, 65, 71, 77-79, 83, 89].	
	Ceropegia purpurascens K.Schum.	H	Roots; Monamela (S)	Boiled and taken orally	Impotence in men [81].	1
	Gomphocarpus fruticosus (L.) W.T.Aiton	SH	Roots; Motsoshapoo (S); Cotton milkweed (E); Firesticks (E); Tontel (A); Tontelbossie (A); Usingalwesalukazi (Z)	Boiled and taken orally	Impotence in men [65, 71, 78, 81].	4
	Hollarhena pubescens Wall. Ex. G.Don	SH	Roots; Fever-pod (E); Khatha-khathane (Tsh)	Decoction drunk twice per day until infection subsides	Gonorrhoea [53].	1
	Hoodia gordonii (Masson)Sweet ex Decne.	SH	Stem bark; Bitterghaap (A); Bobbejaanghaap (A); Jakkalsghaap (A)	Not mentioned	Mouth ulcers associated with HIV-AIDS [43].	1
	Raphionacme hirsuta (E.Mey.) R.A.Dyer	H	Bulb; Tshengwa (S); False gentian (E); Intsema (X); Khadi (A); Khadiwortel (A); Umathangane (Z); Umathanjana (Z)	Not mentioned	Unidentified STIs [19].	1
	Rauvolfia caffra Sond.	T	Roots; Unadzi (TSH); Muraldi (Tsh); Quinine Tree (E); Umhlambamanzi (Z); Umhlambamaze (Z); Umjele (X); Umjelo (X)	Not mentioned	Unidentified STIs [5, 18].	2

	Sarcostemma viminale (L.) R.Br.	C	Roots; Mokwekwere o mogolo (S); Moraro (S)	Not mentioned	opportunistic infections associated with HIV-AIDS [80, 83].	2
	Tabernaemontana elegans Stapf	T	Roots and leaves; Muhatu (Tsh); Umkhadlu (Z); Toad Tree (E)	Roots boiled in water, taken orally	Mouth ulcers associated with HIV-AIDS and gonorrhoea [44, 49, 57].	3
	Wrightia natalensis Stapf.	T	Roots and stem bark; Musunzi (Tsh); Musunzi (Tsh), Saddle-pod (E)	Ground into powder and immersed in water, use as gargle	Mouth ulcers associated with HIV-AIDS and impotence [53, 57].	2
**Araceae**	*Zantedeschia aethiopica* (L.) Spreng.	H	Roots; Onskelk (A); Arum lily (E); Calla lily (E); Hottentotsblare (A), Hottentotsbrood (A), Ihlukwe (Z), Intebe (Z), Intebe (X)	Not mentioned	Chlamydia [49, 75].	2
**Araliaceae**	*Cussonia paniculata* Eckl. & Zeyh. subsp. *paniculata*.	SH	Stem bark and leaves; Bergkiepersol (A); Mountain Cabbage Tree (E)	Powdered plant material applied directly to shingles	Shingles and as immune booster against opportunistic infections associated with HIV-AIDS [9].	1
	Cussonia spicata Thunb.	T	Whole plant; Cabbage Tree (E); Kiepersol (A); Musenzhe (TSH); Umsenge (Z)	Powdered plant material applied directly to shingles	Various unidentified STIs, shingles and as immune booster against opportunistic infections associated with HIV-AIDS [9, 95].	2
**Asclepiadaceae**	*Xysmalobium undulatum* (L.) W.T.Aiton	H	Roots, Ishongwe (Z); Ishinga (Z); Bitterhoutwortel (A); Bitterwortel (A); Iyeza	Boiled and taken orally	Syphilis [39,40].	2

			Elimhlophe (X); Leshokgoa (S)			
**Asparagaceae**	*Agave americana* L.	H	Leaves, American agave (E); American aloe (E); Blougaringboom (E); gareboom (E); kaalgaarboom (A); Lekhala (S)	Sliced and immersed in water, taken orally	Chlamydia, gonorrhoea, and syphilis [51].	1
	Agave sisalana Perrine	SH	Whole plant, Tshikwenga (Tsh); Sekhopha sa dintlo (S); agave (E); hemp plant (E)	Boiled and taken orally	Chlamydia, "makgoma", gonorrhoea, syphilis, and genital warts [50, 51, 60].	3
	Asparagus concinnus (Baker) Kies	SH	Roots, Mphalatsamaru (S); Wild asparagus (E); Haakdoring (A)	Boiled and taken orally	Gonorrhoea [41].	1
	Asparagus densiflorus (Kunth) Jessop	H	Leaves, Doringtou (A); Imbelekazana (Z); Isigoba (Z); Thorny Creeper (E); Mphalatsamaru (S)	Not mentioned	Opportunistic infections associated with HIV-AIDS [9].	1
	Asparagus falcatus L.	H	Roots; Doringtou (a), Imbelekazana (z), Isigoba (z), Thorny Creeper (E); Asparagus fern (E); Gundakhanga (TSH)	Not mentioned	Various unidentified STIs [49].	1
	Drimia alta R.A.Dyer	H	Bulb, Brandui (A); Indongana-zibomvana (Z); Kekanama (S)	Not mentioned	Various unidentified STIs [81].	1
	Merwilla plumbea (Lindl.) Speta	H	Bulbs; Blousalngkop (A); Blue Squill (E); Ichitha (Z); Imbizenkulu (Z);	Burnt applied to affected area	Shingles associated with HIV-AIDS [9, 63].	2

			Inguduza (Z); Kherere (S), Ubulika (X)			
	Sansevieria aethiopica Thunb.	H	Whole plant; Bowstring Hemp (A); Kafferwortel (E); Wildewortel (A)	Not mentioned	Various unidentified STIs [38, 89].	2
**Asphodelaceae**	*Aloe arborescens* Mill.	H	Roots and leaves, Kgopha ya fase (S); Krantz aloe (E); Kransaalwyn (A); Ikalene (X); Inkalane (Z); Umhlabana (Z)	Boiled and taken orally	Opportunistic infections associated with HIV-AIDS [80, 82, 83].	3
	Aloe chabaudii Schönland	H	Roots, Tshikhopha (Tsh); Sekgopha (S); Inhlaba (Z); Inkalane (Z)	Not mentioned	Various unidentified STIs [50, 68].	2
	Aloe falcata Baker	SH	Roots, Kgopha (S); Vanrhynsdorp aloe (A)	Not mentioned	Syphilis and opportunistic infections associated with HIV-AIDS [80, 83].	2
	Aloe ferrox	SH	Leaves and roots; Kgopha (S); Inhlaba (Z)	Not mentioned	Opportunistic infections associated with HIV [9, 64, 96].	3
	Aloe marlothii A.Berger	T	Roots and leaves, Kgopha ya go ema, Seema ka maoto (S); Inhlaba (Z); Umhlaba (Z); Mountain aloe (E); Flat-flowered aloe (E); Bergalwyn (A)	Boiled and taken orally	Gonorrhoea and opportunistic infections associated with HIV-AIDS [44, 45, 52, 65, 80, 83].	6
	Aloe zebrina Baker	H	Roots; Tsikele, Kgophana (S)	Not mentioned	Various unidentified STIs [19].	1
	Bulbine alooides (L.) Willd	H	Leaves; Ibhucu (Z); Kopiva (A); Waterpypie (A); Wildekopiva (A)	Sap is applied directly to the skin	Shingles and oral thrush associated with HIV-AIDS [9].	1

	Bulbine anguistifolia Poelln.	H	Roots; Marumo a ngata (S)	Boiled and taken orally	Impotence in men [19]. (	1
	Bulbine natalensis Bake r	H	Roots and leaves; Geelkopieva (A); Ibhucu (Z); Ibhucu (X); Incelwane (X), Water Glass (E)	Boiled are drunk three times daily until infection subsides	Various unidentified STIs [90, 91].	2
**Asteraceae**	*Ageratum conyzoides* (L.) L.	H	Leaves, Tshidzingambule (Tsh); Goatweed (E); Tropical whiteweed (E)	Immersed in water and taken orally	Boost immunity in HIV-AIDS patients [54].	1
	Ageratum houstonianum Mill.	H	Roots, Munyelenga (Tsh); Blue billygoat weed (E); Bluemink (E)	Not mentioned	Gonorrhoea [60].	1
	Artemisia annua L.	H	Roots, Mohlaswapatla (S); Sweet wormwood (E); Sweet annie (E)	Boiled and taken orally	Impotence in men [46, 78, 81].	3
	Artemisia afra Jacq. ex Willd.	H	Whole plant; African wormwood (E); Wilde-als (A); Umhlonyane (X); Mhlonyane (Z); Lengana (S)	Boiled and taken orally	Impotence in men [65].	1
	Aster bakerianus Burtt Davy ex C.A.Sm.	H	Roots; Noxgxekana (X); Udlatshana (Z); Udlutshana (Z), Umaqhunsula (Z), Umhlungwana (Z)	Boiled and drunk	Unidentified STIs [90, 91].	2
	Athrixia phylicoides DC.	H	Roots, Mutshatshaila (Tsh) Mubosotie (Tsh); Ishanela (Z); Ishayelo (Z); Itshelo	Not mentioned	Impotence in men [50, 91].	2

			(Z); Kaffertee (A); Bush tea (E)			
	Bidens pilosa L.	H	Whole plant, Uqadolo (Z); Blackjack (E)	Boiled and taken orally	Genital sores and genital warts [44].	1
	Callilepis laureola DC.	H	Roots; Ox-eye Daisy (E); Wildemargriet (A); Amafuthomhlaba, Ihlamvu, Impila (Z)	Not mentioned	Various unidentified STIs [65].	1
	Callilepis salicifolia Oliv.	H	Tuber, Phelana (S); Ox-eye daisy (E); Wildemargriet (A); Amafuthomhlaba (E); Ihlamvu (E); Impila (Z)	Not mentioned	Gonorrhoea and opportunistic infections associated with HIV-AIDS [45, 80, 83].	3
	Dicoma anomala Sond.	H	Tuber, Fever bush (E); Stomach bush (E); Maagbitterwortel (A); Hloenya (S); mohlasetse (S); inyongana (X); Isihlabamakhondlwane (Z); Umuna (Z)	Boiled and taken orally	Various unidentified STIs [82].	1
	Geigeria aspera Harv.	H	Whole plant, Makgonatsohle (S); Misbekvermeerbos (A); Vermeerbos (A)	Not mentioned	Opportunistic infections associated with HIV-AIDS [80, 83].	2
	Helichrysum caespititium (DC.) Sond. ex Harv.	H	Whole plant, Matsana (S); Boriba (S), Botsiki-nyane (S); Lelula-phooko (S); Speelwonderboom (A)	Not mentioned	Gonorrhoea [45, 65, 80, 83].	4
	Helichrysum kraussii Sch.Bip.	H	Roots, Tshifulathulo (Tsh); Isipheshane (Z); Isiqoqo	Not mentioned	Impotence in men [50].	1

			(Z); Sewejaartjie (A); Straw everlasting (E)			
	Kleinia longifolia Haw.	H	Roots, Lekgabolo (S); Chimanimani kleinia (E); Chimanimani-kleinia (A)	Not mentioned	Various unidentified STIs and chlamydia [56, 80, 83].	3
	Pechuel-loeschea leubnitziae (Kuntze) O.Hoffm.	SH	Roots; Bitterbossie (A); Stinkbossie (A); Stinkbush (E)	Powdered plant material licked by tongue.	Impotence in men [53].	1
	Platycarpha glomerata (Thunb.)	H	Leaves and roots; Usiphahluka (Z)	Not mentioned	Various unidentified STIs associated with females and "Isipatsholo" [62, 64].	2
	Schkuhria pinnata (Lam.) Kuntze ex Thell.	H	Whole plant; Luswielo (Tsh)	Boiled and taken orally	Gonorrhoea and opportunistic infections associated with HIV-AIDS [54, 57].	2
	Senecio serratuloides DC.	H	Whole plant, Unsukumbili (Z); Ichazampukane (Z); Two-day Cure (E); Umaphozisa (Z); Umkhuthelo (Z)	Boiled, taken orally	Various unidentified STIs and opportunistic infections associated with HIV-AIDS [44, 75].	2
	Spilanthes acmella (L.) L.	H	Whole plant, Tshishengelapofu (Tsh); Toothache plant (E); Paracress (E).	Powdered and soaked in water, taken orally	Various unidentified STIs [57].	1
	Tagetes minuta L.	H	Leaves, Mushashthuri (Tshiv); khaki bush (E); khaki weed (E) African marigold (E); Kakiebos (A); khakibos (A); Mbanje (ND)	Dried leaves are boiled in water, taken orally	Mouth ulcers associated with HIV-AIDS [57].	1

	Tarchonanthus camphoratus L.	SH	Leaves, gqeba-elimhlophe (Z); Isiduli-sehlathi (Z); Kleinvaalbos (A); Mathola (X), Mofahlana (S); Moologa (Tsh)	Leaves infusion is inhaled	Various unidentified STIs [38, 89].	2
**Balanophoraceae**	*Sarcophyte sanguinea* Sparrm.	P	Stem, Umavumbuka (Z); Ihlule (Z); Umavumbuka (Z); Wolwekos (A)	Boiled, taken orally	Gonorrhoea and opportunistic infections associated with HIV-AIDS [44].	1
**Basellaceae**	*Anredera cordifolia* (Ten.) Steenis	C	Tuber and seeds; Bridal wreath (E); Cascade creeper (E)	Crushed and boiled, taken orally	Chlamydia, gonorrhoea, syphilis and genital warts and opportunistic infections associated with HIV-AIDS [5, 51, 71].	3
**Bignoniaceae**	*Jacaranda mimosifolia* D.Don	T	Stem bark and leaves; Mudzhagarannnda (Tsh)	Crushed and boiled, taken orally	Gonorrhoea [51, 60].	1
	Kigelia africana (Lam.) Benth.	T	Pods, leaves and Stem bark, Umvongothi (Z)	Boiled and taken orally	Syphilis, opportunistic infections associated with HIV-AIDS [44, 51, 95, 90, 91].	5
**Bombaceae**	*Adansonia digitata* L.	T	Stem bark, Muvhuyu (Tsh)	Not mentioned	Various unidentified STIs [50, 68-70].	4
**Brassicaceae**	*Boscia albitrunca* (Burch.) Gilg & Benedict	T	Roots, Mohlopi (S); Caper bush (E); Coffee tree (E); Koffie (A); Groot-witgat (A); Koffieboom (A); Umpunzito (X); Umtopi (ND); Isinama (Z); Inyokiziphinda (Z).	Boiled and taken orally	Opportunistic infections associated with HIV-AIDS [80, 82, 83].	3

	Maerua edulis (Gilg & Gilg-Ben.) DeWolf	SH	Roots, Mutshalimela (TSH); Sozwe Tree (E); Bush-cherry (E); Inswaniswani (Z)	Not mentioned	Various unidentified STIs [76].	1
**Burseraceae**	*Commiphora africana* (A.Rich.) Engl. Var *africana*	SH	Roots; Hairy Corkwood (E); Harige Kanniedood (A); Iminyela (Z); Morôka (S); Mothapô (S); Ozidlaxo (Z), Poison-grub Corkwood (E)	Not mentioned	Impotence in men [53].	1
	Commiphora merkeri Engl.	SH	Roots, Mutonyombidi (Tsh); Ringbas-kanniedood (A); Zebra tree (E); Zebra-bark Corkwood (E)	Crushed and boiled, taken orally	Impotence in men [50].	1
	Commiphora viminea Burtt. Davy	T	Roots; Mutonyombidi (Tsh); Ringbas-kanniedood (A); Zebra Tree (E); Zebra-bark Corkwood (E); Zebrabas-kanniedood (A)		Impotence [53].	1
**Cactaceae**	*Opuntia ficus-indica* (L.) Mill.	SH	Roots, Motloro, Tlorofeile (S); Mudoro (Tsh); Barbary fig (E); Cactus pear (E); Prickly pear (E)	Boiled and taken orally	Various unidentified STIs, chlamydia, gonorrhoea, syphilis, gonorrhoea, "makgoma", shingles arising from HIV-AIDS and drop [19, 45, 50-53, 65, 78, 79, 82, 83].	11
	Opuntia stricta (Haw.) Haw.	SH	Roots and stem, Umdolofiya (Z); Tlorofeile (S); Mudoro (Tsh)	Boiled, taken orally	Gonorrhoea and genital wards [44, 51].	2

**Canellaceae**	*Warburgia salutaris* (G.Bertol.) Chiov.	T	Roots and stem bark, Mulanga (Tsh); Manakha (Tsh); Isibaha (Z); Amazwecehlabayo (Z); Molaka (S); Fever tree (E); Koorsboom (A); Peperbasboom (A)	Boiled, taken orally	Various unidentified STIs, impotence and mouth ulcers associated with HIV-AIDS [49, 50, 57, 76, 90, 91, 94	7
**Cannabaceae**	*Cannabis sativa* L. var. *indica* (Lam.) Wehmer	H	Whole plant; Dagga (E); Lebake (S); Patse (S)	Boiled and taken orally	Immune booster for HIV-AIDS patients [90].	1
**Cappariaceae**	*Capparis tomentosa* Lam.	SH	Intsihlo (X); Inkunzi-ebomvu (Z); Umqoqolo (Z); Kapperbos (A); Woolly Caper Bush (E); Woolly Caper-bush (E)	Infusion, steam bath	"*Nta shete*" [39, 40, 49].	3
	Cladostemon kirkii (Oliv.) Pax & Gilg	T	Roots, Isidumbu (Z); Umthekwini (Z); iyamuka (Z); Drievingerbos (A); Three-finger bush (E)	Decoction taken orally	Various unidentified STIs [44].	1
**Caricaceae**	*Carica papaya* L.	T	Roots and fruits, Mophoophoo (S), Mupapawe (Tsh), Upopo (Z)	Roots boiled and taken orally; Unripe fruits are squeezed for impotence	Various unidentified STIs, gonorrhoea and impotence [44-46, 50, 56, 59, 65, 78, 79, 81, 83] Roots may also be used to treat oral candidiasis [57].	12
**Caryophylaceae**	*Krauseola mosambicina* Pax & Hoffm.	H	Whole plant, Isihlaza (Z)	Decoction taken orally	Unidentified STIs and sores [44].	1
**Casuarinaceae**	*Casuarina cunninghamiana* Miq.	T	Flowers, Musanana (Tsh); River Sheoak (E); Origin Vic (E)	Not mentioned	Gonorrhoea [60	1

**Crassulaceae**	*Bryophyllum delagoense* (Eckl. & Zeyh.) Druce	SH	Leaves, Mutungupfa (Tsh)	Not mentioned	Gonorrhoea [60].	1
	Bryophyllum pinnatum (Lam.) Oken	SH	Leaves, Umvuthi (Z); Mother-in-law (E); Leaf of Life (E); Wonder of the world (E)	Boiled and taken orally	Gonorrhoea [44].	1
**Celestraceae**	*Cassine transvaalensis* (Burtt Davy) Codd	T	Stem bark; Mulumanamana (TSH); Mukuvhazwivhi (TSH); Transvaal saffron (E); Umgugudo (Z)	Not mentioned	Various unidentified STIs [76].	1
	Catha edulis (Vahl) Endl.	SH	Roots, Lehwane (S); Mohlatse (S); Bushman's Tea (E); Igqwaka (X), Inandinandi (ND); Khat (E); Khat (A); Luthadzi (Tsh); Lwani (Tsh);	Boiled and taken orally	Impotence [63, 81].	2
	Elaeodendron transvaalense (Burtt Davy) R.H.Archer	T	Stem bark and roots, Monamane (S), Mukuvhazwivhi, Mulumanamana (Tsh)	Boiled and taken orally and as mouth wash	Various unidentified STIs and opportunistic infections associated with HIV-AIDS, oral thrush [5, 19, 50, 53, 58, 75, 80, 82].	8
	Gymnospora senegalensis (Lam.) Loes.	H	Root bark and leaves; Mophato (S); Rooidoring (A); Rooi-pendoring (A); Tshibavhe (Tsh); Tshiphandwa (Tsh); Ubuhlangwe (Z)	Boiled and taken orally	Management of opportunistic infections associated with HIV-AIDS and impotence [19, 81].	2

	Hippocratea longipetiolata Oliv.	SH	Roots, Mutshaliri (Tsh)	Boiled and taken orally	Opportunistic infections associated with HIV-AIDS, oral thrush [57].	1
**Clusiaceae**	*Garcinia livingstonei* T.Anderson	SH	Roots; Muphiphi (Tsh); African Mangosteen (E); Lowveld Mangosteen (E); Wild Plum (E); Umphimbi (Z)	Not mentioned	Impotence men [50, 53, 95].	3
**Combretaceae**	*Combretum caffrum* (Eckl. & Zeyh.) Kuntze	T	Roots; Cape Bushwillow (E); Rooiblaar (A); Rooiblad (A); Umdubu (Z); Vaderlandwilgerboom (A)	Not mentioned	Unidentified STIs [91].	1
	Combretum molle R.Br. ex G.Don	T	Leaves and roots, Umbondo (Z); Mohwelere (S); Mugwiti (Tsh), Velvet bushwillow (E)	Boiled and taken orally	Various unidentified STIs [44, 49].	2
	Combretum paniculatum Vent.	T	Roots, Mukopokopo (Tsh)	Not mentioned	Various unidentified STIs [49, 50].	2
	Combretum vendae A.E .van Wyk var.*vendae*	SH	Roots, gopokopobani (TSH); lugwiti(TSH); Vendabushwillow (E); Vendaboswilg(A)	Dried roots are pounded and licked.	Impotence in men [53].	1
	Terminalia prunioides M.A.Lawson	T	Stem bark; Hardekoolboom (A); Lowveld Cluster-leaf (E); Lowveld Terminalia (E); Mutsiara (S); Mutwari (Tsh)	Not mentioned	Various unidentified STIs [57].	1

	Terminalia sericea Burch ex DC.	T	Stem bark and roots; Monakanakane (S); Imkhonono (Z); Assegai Wood (E); Bloubos (A); Bosvaalbos (A); Moxonono (S); Mususu (TSH), Sand Yellow Wood (E)	Boiled and taken orally	Various unidentified STIs, impotence and opportunistic infections associated with HIV-AIDS including internal and external sores [5, 19, 38, 41, 44, 50, 53, 65, 89].	9
**Commelinaceae**	*Commelina eckloniana* Kunth	H	Whole plant, Umkhonto (Z)	Boiled and drunk until infection subsides	Gonorrhoea [62, 64].	2
	Cyanotis speciosa (L.f.) Hassk.	H	Whole plant; Ingonga (Z); Inkombo (z); Insonga (z); Job's Tears (E); Ndou Muhulu (Tsh); Umagoswana (X); Umakhothigobile (Z); Wandering Jew (E)	Boiled and taken orally	Gonorrhoea [62, 64].	2
**Convolvulaceae**	*Ipomoea batatas* (L.) Lam.	H	Leaves; Ubhatata (Z)	Boiled and taken orally	Gonorrhoea [44].	1
	Ipomoea bolusiana Schinz	H	Bulb; Mokutu (S); Seakhoe (S); Narrow-leaved pink Ipomoea (E); Morning glory (E)	Boiled and taken orally	Various unidentified STIs [19, 50, 67].	3
	Ipomoea crassipes Hook.	H	Bulb; Mothokhoana (S); Sekutle (S); Seletjane-se-setona (S); Ubhoqo (Z); Uvimbukhalo (Z);	Not mentioned	Opportunistic infections associated with HIV-AIDS [87].	1

			Wildepatata (A); Wildewinde (A)			
	Ipomoea pellita Hallier f.	H	Tubers; Ubhoqo (Z)	Not mentioned	Impotence [62, 64].	2
	Ipomoea obscura (L.) Ker Gawl.	H	Bulb; Kgomodimaswi (S); Usiboniseleni (Z); Wild petunia (E); Wildepatat (A)	Not mentioned	Gonorrhoea [44, 80, 83].	3
**Crassulaceae**	*Bryophyllum delagoense* (Eckl. & Zeyh.) Druce	SH	Leaves, Mutungupfa (Tsh)	Not mentioned	Gonorrhoea [60].	1
	Bryophyllum pinnatum (Lam.) Oken	SH	Leaves, Umvuthi (Z); Mother-in-law (E); Leaf of Life (E); Wonder of the world (E)	Boiled and taken orally	Gonorrhoea [44].	1
	Crassula muscosa L.	H	Stems and leaves; Lizard's Tail (E)	Decoction	Mouth ulcers associated with HIV-AIDS [43].	1
**Cucurbitaceae**	*Cucumis myriocarpus* Naudin	H	Roots, Magapyana (S); Wild Cucumber (E); Gooseberry cucumber (E); Sendelenja (Z); Small wild Melon (E); Streepwildekomkommer (A); Wildekomkommer (A)	Boiled and taken orally	"Tshofela" and gonorrhoea [45, 79, 83].	3
	Cucumis zeyheri Sond.	H	Fruit, Monyaku; Wild cucumber (E); itterappel (A); Inhlakahlela (Z); Wild cucumber (E); Wilde-agurkie (A)	Immersed in water and used as enema	Chlamydia [59].	1

	Citrullus lanatus (Thunb.) Matsum. & Nakai	H	Roots; Morotse (S); Kaffir Watermelon (E); Kakoer (A); Koer Koer (A); Koerkoer	Boiled and taken orally	Gonorrhoea and opportunistic infections associated with HIV [65, 79, 83, 94].	4
	Kedrostis nana (Lam.) Cogn. var. nana	C	Tubers, Bryony (E); Ystervarkpatats (A)	Not mentioned	Gonorrhoea, Hepatitis B and syphilis [91, 94].	2
**Cupressaceae**	*Widdringtonia cupressoides* (L.) Endl.	SH	Roots; Thaululo (Tsh)	Not mentioned	Various unidentified STIs [50].	1
**Curtisiaceae**	*Curtisia dentata* (Burm.f.) C.A.Sm.	T	Stem bark, Modulatshwene (S); Asgaaiboom (A); Assegaai (E); Injundumlahleni (Z); Mufhefhera (Tsh); Musangwe (Tsh); Umguna (X); Umgxina (X); Unhlebe (X); Unlahleni (X); Uphephelelangeni (Z);	Boiled and taken orally	Various unidentified STIs, impotence [47, 48, 90].	3
**Dioscoreaceae**	*Dioscorea sylvatica* Eckl.	H	Bulb; Setagwa (S); Isidakwa (Z); Forest Elephant's Foot (E)	Not mentioned	Gonorrhoea [45, 80, 82].	3
**Dipsacaceae**	*Scabiosa columbaria* L.	H	Whole plant, Bitterbos (A); Hlako-ya-pitsi (S); Ibheka (Z); Ilelemimoya (X); Ubucubele (Z), Udoloqina (Z)	Ointment from roots is applied to wounds	Syphilis and gonorrhoea [38, 89].	2
**Dracaenaceae**	*Sansevieria hyacinthoides* (L.) Druce	H	Roots; Makgotse (S); Aambeiwortel (A); Isikholokotho (Z); Isikholokotho (X);	Not mentioned	Gonorrhoea [80, 83].	2

			Isikhwendle (Z); Isitokotoko (Z); Mother-in-law's-tongue (E), Skoonma-se-tong (A), Wildedatel (A)			
	Sansevieria trifasciata P rain	H	Roots, Devils tongue (E); Snakees tongue (E); Snake plant (E)	Bioled and taken orally	Opportunistic infections associated with HIV-AIDS [65].	1
**Ebenaceae**	*Diospyros lycioides* subsp. guerkei (Kuntze) De Winter	T	Roots, Bessieboom (A); Jakkalsbessie (A); Muthala (Tsh); Quilted bluebush (E)	Not mentioned	Various unidentified STIs [53, 75, 87].	3
	Diospyros mespiliformis Hochst. ex A.DC.	T	Fruit, Musuma (Tsh); African Ebony (E); Ebbehout (A); Musuma (Tsh); Transvaalebbehout (A); Umdhlausu (ND), Umdlawuzo (ND)	Crushed and mixed with water, taken orally.	Various unidentified STIs, impotence, oral thrush associated with HIV-AIDS [57, 84, 86, 88].	4
	Diospyros whyteana (Hiern) P.White	T	Leaves, Munya Vhili (Tsh); African bladder nut (E); Black bark (E); Bostolbos (A); Kraaibessie (A)	Raw leaves are immersed in water to make tea, Used as douche	Oral thrush associated with HIV-AIDS [57].	1
	Euclea crispa (Thunb.) Gürke	T	Roots, Mokwerekwere (S); Blue guarri (E); Bosghwarrie (A); Idungamuzi (Z); Mungule-nyele (Tsh); Munyele (Tsh)	Not mentioned	Opportunistic infections associated with HIV-AIDS [65, 80, 82, 83].	4

	Euclea natalensis A.DC.	T	Roots, Mohlakola,(S); Mokgokgono (S); Large-leaved guarri (Eng.); umKhasa (X); inKunzi-emnyama (Z), umHlalanyamazane,(Z).	Boiled and taken orally	Various unidentified STIs, gonorrhoea [38, 41, 50, 61, 88].	5
**Euphorbiaceae**	*Alchornea laxiflora* Pax & K. Hoffm.	SH	Roots; Lowveld Bead-string (E); Murunda-malofha (Tsh); Venda Bead-string (E); Venda-kralesnoer (A)	Not mentioned	Various unidentified STIs [53].	1
	Croton gratissimus Burch.	SH	Bulb, Mafunyungule (Tsh); Mookgwasane (S); Mufhorola (Tsh); Muthatha-khubi (Tsh); Rekstokbos (A); Stinkhout (A); Uhubeshane (Z)	Crushed, soaked into hot water and drunk	Gonorrhoea, syphilis, Oral thrush associated with HIV-AIDS [38, 57, 82].	3
	Euphorbia hypericifolia L.	SH	Whole plant, Umaphipha (Z); Graceful spurge (E)	Boiled and taken orally	Gonorrhoea [44, 72].	2
	Euphorbia maleolens E.Phillips	H	Whole plant, Rofa-bja-tau (S)	Boiled and taken orally	Opportunistic infections associated with HIV-AIDS [65, 80, 83].	3
	Euphorbia tirucalli L.	SH	Roots, Umnduze (Z); Motlalamela (S); Mutungu (Tsh); Rubber euphorbia (E); Umhlontlo (X); Umnduze (Z); Umsululu (Z)	Boiled and taken orally	Gonorrhoea and impotence [44, 61].	2
	Jatropha curcas L.	SH	Roots and leaves; Sehlare sa banna (S); Mokhure (S);	Boiled and taken orally	Opportunistic infections associated with HIV-AIDS	1

			Barbados nut (E); Bubble bush (E)		including vaginal candidiasis [81].	
	Jatropha erythropoda Pax & K.Hoffm.	H	Bulb, Thotamadi (S); Rooikambroo (A)	Boiled and taken orally	Chlamydia [59].	1
	Jatropha zeyheri Sond.	H	Bulb, Sefapabadia (S); Ugodide (Z), Verfbol (A)	Boiled and taken orally	Various unidentified STIs, gonorrhoea and "divhu" [19, 45, 53, 65, 66, 80, 83].	7
	Ricinus communis L.	SH	Whole plant; Mokhure (S); Mupfure (Tsh); Castor oil (E)	Boiled and taken orally	Chlamydia, gonorrhoea, syphilis, "makgoma" and opportunistic infections associated with HIV-AIDS [51, 52, 60].	3
	Tragia rupestris Sond.	H	Roots; Brandneuker (A); Imbabazane (Z)		Impotence [53].	1
**Fabaceae**	*Albizia adianthifolia* (Schum.) Wight	T	Leaves, Igowane (Z); Umbhelebhele (Z); Umgadankawu (Z); Flat-crown (E); Isicangca (X); Muelela (Tsh); Muomba-ngoma (Tsh)	Decoction taken as an enema	Syphilis and impotence [44, 53].	2
	Albizia anthelmintica Brongn.	SH	Roots, Muime (Tsh); Arub (A); Bonthout (A); Cherry-blossom tree (E); Mmola (S); Muime (Tsh); Mukuvhavhadinda (Tsh); Umnala (Z); Umnalahanga (Z)	Not mentioned	Various unidentified STIs [50].	1
	Albizia gummifera (J.F.Gmel.) C.A.Sm.	T	Stem bark; Umgandakawu (Z)	Boiled and taken orally	Various unidentified STIs []. 39, 40].	1

	Albizia versicolor Oliv.	T	Stem bark; Muvhambabgoma (Tsh); Large-leaved false-thorn (E); Mmola (S); Mohlabafota (S); Mohlalabata (S); Mutamba-pfuna (Tsh), Umphisu (Z); Umvangazi (Z)	Not mentioned	Various unidentified STIs, impotence in men [49, 50].	2
	Bauhinia galpinii N.E.Br.	SH	Roots; Mohohoma (S); Mutswiriri (Tsh)	Not mentioned	Various unidentified STIs, impotence [19, 53, 54].	3
	Bolusanthus speciosus (Bolus) Harms	SH	Roots and stem; Mukambana (Tsh)	Boiled and taken orally	Various unidentified STIs and impotence [50, 53, 68-70].	5
	Burkea africana Hook.	T	Roots and seeds; Monatlo (S), Mufhulu (Tsh)	Both roots and seeds are ground and boiled and drunk	Opportunistic infections associated with HIV-AIDS [53, 57, 58, 80, 83].	5
	Caesalpinia decapetala (Roth) Alston.	SH	Roots; Mokgabane (S)	Boiled and taken orally	Impotence and gonorrhoea [45, 53, 65, 78, 79, 83].	6
	Cassia abbreviata Oliv.	SH	Roots and stem bark; Monepenepe, Molomanama (S)	Boiled and taken orally	Various unidentified STIs, chlamydia, impotence, and as immune booster for HIV-AIDS patients [19, 41, 53,59, 74, 84].	6
	Colophospermum mopane J. Kirk. ex Benth.) Leonard	T	Roots; Mopane (S)	Boiled and taken orally	Impotence [53].	1
	Dalbergia melanoxylon Guill. & Perr.	T	Stem bark; African Blackwood (E); African Ebony (E);	Not mentioned	Various unidentified STIs [49].	1

			Driedoringebbehout (A); Ebbehout (A); Ebony (E); Grenadille Wood (E); Muuluri (Tsh)			
	Dichrostachys cinerea L.	SH	Roots; Moretshe (S)	Not mentioned	Impotence and syphilis [53, 91].	2
	Elephantorrhiza burkei Benth.	H	Roots, Mohauwane wa thaba, Mositsane (S), Tshisevhufa (Tsh)	Applied to infected areas of the skin, decoction drunk	Various unidentified STIs, chlamydia, aphrodisiac, opportunistic infections associated with HIV-AIDS [19, 50, 53, 57, 59, 66, 68-70, 80-83].	11
	Elephantorrhiza elephantina (Burch.) Skeels	H	Roots, Mohauwane, Mosehlana, Mositsane (S)	Boiled and taken orally	Syphilis, chlamydia, impotence in men [19, 59, 66, 75, 81-83, 91].	8
	Erythrina caffra Thunb.	SH	Roots, Umsisnsi	Boiled and taken orally	Various unidentified STIs and genital warts [38, 44].	2
	Eriosema cordatum E.Mey.	H	Roots; Leshetla (S); Ubangalala (Z); Umhlabankunzi (Z); Uqonsi (Z); Uqontsi (Z)	Not mentioned	Impotence in men [61].	1
	Erythrina lysistemon Hutch.	T	Stem bark; Muvhale (Tsh)	Boiled and taken orally	Impotence in men [54]. Ground roots and stem bark boiled and used as mouthwash for oral candidiasis [57].	2
	Faidherbia albida (Delile) A.Chev.	T	Stem bark; Muhoto (S)	Not mentioned	Various unidentified STIs and impotence for men [50, 53].	2
	Mundulea sericea (Willd.) A.Chev.	SH	Roots; Maibana, Mohato, Mositatlou (S); Mukandandou (Tsh)	Not mentioned	Impotence for men [50, 53].	2

	Ormocarpum trichocarpum (Taub.) Engl.	T	Roots; Caterpillar Bush (E); Caterpillar Pod (E); Hairy Caterpillar Pod (E); Isithibane (Z); Umsindadlovana (Z); Mosepe (S); Mugogodwane (Tsh), Muthari (Tsh); Rusperboontjie (A)	Not mentioned	Impotence in men [88].	1
	Peltophorum africanum Sond.	T	Roots and stem bark; Mosehla (S); Musese (Tsh)	Boiled and taken orally	Various unidentified STIs, impotence and skin infections associated with HIV-AIDS [19, 44, 49, 50, 57, 65, 66, 68, 75, 76, 80-83].	14
	Piliostigma thonningii (Schum.) Milne-Redh.	T	Roots; Mukolokote (Tsh); Camel's Foot (E); Monkey Bread (E); Picture-frame Tree (E); Rhodesian Bauhinia (E); Kameelspoor (A); Mokgôrôpô (S); Rhodesiese Bauhinia (A)	Not mentioned	Various unidentified STIs and impotence [50, 88].)	2
	Philenoptera bussei (Harms) Schrire	T	Roots; Mphata (S)	Boiled and taken orally	Chlamydia [59].	1
	Philenoptera violacea (Klotzsch) Schrire	T	Roots; Apple-leaf (E)	Not mentioned	Impotence in men [53].	1
	Pterocarpus angolensis DC.	T	Stem bark; Mutondo (Tsh)	Not mentioned	Various unidentified STIs and "*divhu*" [50, 53, 68].	3
	Pterocarpus rotundifolius (Sond.) Druce	T	Stem bark, Muataha (Tsh)	Boiled and taken orally	Oral candidiasis associated with HIV-AIDS [57].	1

	Schotia brachypetala Sond.	T	Roots and stem bark; Mununzu (Tsh); African Greenheart (E); African Walnut (E); Umgxamu (Z); Uvovo (Z); Uvovovo (Z)	Boiled and taken orally	Opportunistic infections associated with HIV-AIDS [57].	1
	Senegalia ataxacantha DC.	T	Roots; Muluwa (Tsh); Flame Acacia (E); Flame Thorn (E); Kaffer-wag-'n-bietjie (A); Kafferwortel (A); Mogokare (S), Ubophe (Z); Ugagane (Z), Umnga (X)	Not mentioned	Impotence in men [50].	1
	Senegalia caffra (Thumb) Wild.	T	Leaves, Morobadiepe (S), Murhovhambado (Tsh); Muvunda-mbado (Tsh); Morobadiepe (S); Umnyamanzi (X); Umthole (X); White thorn (E)	Leaves are dried, burned and mixed with animal fat, applied to mouth blisters and ulcers.	Opportunistic infections associated with HIV including mouth ulcers [57].	1
	Senna auriculata (L.) Roxb.	T	Leaves, Muduwishango (Tsh)	Fresh leaves are crushed and drunk orally	Opportunistic infections associated with HIV-AIDS [57].	4
	Senna didymobotrya (Fresen.) H.S.Irwin & Barneby	SH	Roots, African senna (E); popcorn senna (E); candelabra tree (E); peanut butter cassia (E)	Crushed and boiled, taken orally	Chlamydia, gonorrhoea, syphilis, impotence, and genital warts [51-53, 76].	3
	Senna italica subsp. *arachoides* (Burch.) Lock	H	Roots, Morotelatshotshi, Setlommana, Mankgane (S)	Boiled and taken orally	Various unidentified STIs, impotence, gonorrhoea, "*makgoma*" and opportunistic infections associated with	9

					HIV-AIDS vaginal candidiasis [41, 45, 59, 80, 81, 83, 88].	
	Senna occidentalis L.	SH	Roots, Modulabadimo (S)	Not mentioned	Impotence in men [53].	1
	Senna petersiana (Bolle) Lock	SH	Seeds, Munembenembe (Tsh); Apiespeul (A); Bohlôko (S); Dwarf Cassia (E); Monkey pod (E), Uhwabile (Z); Umnembenembe (Z)	Dried seeds are ground into powder and boiled in water	Impotence, gonorrhoea, and opportunistic infections associated with HIV-AIDS [5, 50, 53, 57, 71, 76, 87].	7
	Sutherlandia frutescens (L.) R.Br.	SH	Leaves; Mokgoroma (S)	Boiled and taken orally	Opportunistic infections associated with HIV-AIDS [9]. (	1
	Tephrosia zoutpansbergensis Bremek.	SH	Roots, Motswaing (S)	Not mentioned	Impotence in men [53].	1
	Vachellia karroo (Hayne) Banfi & Glasso	T	Roots and stem bark, Mooka (S), Muunga (Tsh); Sweet thorn (E); Soetdoring (A); UmuNga (Z)(X)	Boiled, used as mouth wash, or applied directly to the genitals to treat vaginal ulcers.	Various unidentified STIs, impotence, opportunistic infections associated with HIV vaginal candidiasis and ulcers [42, 50, 55, 53, 57, 68-70, 87, 88, 90, 95].	12
	Vachellia permixta Burtt Davy	SH	Roots, Moselaphala (S); Doringboom (A); Fyndoring (A); Hairy Acacia (E); Hairy Thorn (E); Mimosa (E)	Decoction taken orally	Chlamydia [59].	1
	Vachelia robusta (Burch.) Kyal. & Boatwr. susbs. *Robusta*	T	Stem bark and roots; Brack Thorn (E); Brakdoring (A); Enkeldoring (A); Moga (S); Narrow-pod Robust	Not mentioned	Impotence in men [53].	1

			Thorn (E); River Thorn (E); Umngamanzi (Z); Umngampunzi (X)			
**Flacourtiae**	*Trimeria grandifolia* (Hochst.) Warb.	T	Leaves, Xidengane (Tsh); Grootblaarysterhout (A); Idlebelendlovu (Z); Mufhanza (Tsh); Muhasha-phande (Tsh), Tshilaphithi (Tsh), Wild Mulberry (E)	Fresh leaves chewed	Mouth ulcers associated with HIV-AIDS [57].	1
**Gentianaceae**	*Anthocleista grandiflora* Gilg.	T	Stem bark; Big Leaf (E); Cabbage Tree (E); Fever Tree (E); Forest Big leaf (E); Grootblaarboom (A); Mueneene (Tsh)	Not mentioned	Impotence in men [53].	1
**Geraniaceae**	*Geranium incanum* Burm.f. var. *incanum*	H	Whole plant; Bloubos (A); Blouganna (A)	Boiled and taken orally	General STIs [90, 91].	2
	Monsonia angustifolia E . Mey. ex A. Rich.	H	Roots; Alsbos (A); Crane's Bill (E); Malengoana (S); Malva Naaldbossie (A)	Not mentioned	Impotence [63].	1
**Grassulaceae**	*Cotyledon orbiculata* L.	SH	Roots, Tsebe ya kolobe (S); Beesbul (A); Pig's ears (E)	Not mentioned	Gonorrhoea [45, 80, 96].	3
**Gunneraceae**	*Gunnera perpensa* L.	SH	Roots, Iphuzi (Z); Imfeyesele (Z); Iphuzi lomlambo (X); Qobo (S); River pumpkin (E); Rivierpampoen (A)	Boiled and taken orally	Gonorrhoea, syphilis [39, 40].	2
**Hernandiaceae**	*Gyrocarpus americanus* Jacq. subsp. *africanus* Kubitzki	T	Roots; Helikopterboom (A), Mudzhoudzhou	Not mentioned	Various unidentified STIs [49].	1

			(Tsh); Mutama (Tsh); Propeller Tree (E)			
**Hyanthaceae**	*Bowiea volubilis* Harv.	H	Bulbs; Umagaqana (X); climbing onion (E); knolklimop (A); ugibisisila (Z)	Applied on infected areas	Syphilis [38-40, 89].	3
	Drimia elata Jacq.	H	Bulbs; Brandui (A); Isiklenama (Z); Jeukbol (A); Jeukui (A); Umqumba (Z); Undongana-zibomvana (Z)	Boiled and taken orally three times a day	Gonorrhoea [45, 83].	2
	Drimia sanguinea (Schinz) Jessop Synonym *Urginea sanguinea* Schinz	H	Bulb, Sekanama (S); Rooislangkop (A)	Boiled and taken orally	Various unidentified STIs, chlamydia and opportunistic infections associated with HIV-AIDS [19, 59, 63,75, 80].	5
	Eucomis autumnalis (Mill.) Chitt.	H	Bulb, Pineapple Lily (E); krulkoppie (A); ubuhlungu becanti (Z); isithithibala esimathunzi (X); umathunga (Z)	Not mentioned	Various unidentified STIs, syphilis and impotence (Van Wyk et al., 2005; Rankoana, 2012; Van Wyk et al., 2013; Rasethe et al., 2019)	4
	Eucomis pallidiflora Baker subsp. *pole-evansii* (N.E.Br.) Reyneke ex J.C.Manning	H	Bulb; Mathubadifala; Giant pineapple lily (E).	Boiled and taken orally	Various unidentified STIs, Chlamydia, gonorrhoea, and impotence [46, 51, 63, 65, 75, 80, 81, 83].	8
	Ledebouria ovatifolia (Baker) Jessop	H	Bulbs; Icubudwana (X); Flat-leaved African hyacinth (E); icubudwana	Boiled and taken orally	General STIs [39].	1

			(Z); Untanganazibomvu (X)			
	Merwilla plumbea (Lindl.) Speta	H	Bulbs; Blousalngkop (A); Blue Squill (E); Ichitha (Z); Imbizenkulu (Z), Inguduza (Z)	Not mentioned	Impotence in men [64, 65].	2
**Hydnoraceae**	*Hydnora africana* Thunb.	P	Fruit; Baviaanskos (A); Idolo-lenkonyane (X); Jackal Food (E); Ubuklunga (X); Umavumbuka (Z)	Over ripe fruit eaten	Mouth ulcers associated with HIV-AIDS [43].	1
**Hypoxidaceae**	*Hypoxis haemerocallidea* Fisch., C.A.Mey.& Avé-Lall.	H	Bulb; Monna wa maledu, Thitikwane (S)	Boiled and taken orally	Used for impotence in men, treat gonorrhoea and immune booster for HIV-AIDS patients [9, 42-45, 53, 75, 80, 81, 83, 92].	11
	Hypoxis latifolia Hook.	H	Roots, Ilabathela (X)	Steam bath and boiled and taken orally	"Nta shete [39].	1
	Hypoxis obtusa Burch. ex Ker Gawl.	H	Bulb, Monna wa maledu, Thitikwane (S)	Not mentioned	Various unidentified STIs, impotence and opportunistic infections associated with HIV-AIDS [46, 56, 63, 65, 80, 83].	6
**Icaninaceae**	*Pyrenacantha kaurabassana* Baill.	H	Roots, Inzema (Z)		Genital warts [44].	1
**Iridaceae**	*Gladioulus dalenii* Van Geel	H	Roots; African Gladiolus (E); Isidwi Esibomvu (Z); Natal Lily (E); Papegaai Gladiolus (A);	Cooked with milk and drunk	Impotence [62, 64].	2

			Udwendweni (Z); Uhlakahle (Z); Wildeswaardlelie (A)			
**Juncaceae**	*Juncus kraussii* Hochst	H	Roots; Incema (Z); Matting Rush (E); Rushes (E)	Not mentioned	Various unidentified STIs in women [62, 64].	2
	Juncus lomatophyllus Spreng	H	Roots; Juncus (E)	Not mentioned	Various unidentified STIs in women [62, 64].	2
**Lauraceae**	*Cinnamomum verum* J.Presl	T	Roots; Siina (S)	Boiled and taken orally	Mouth ulcers and skin fungal infections associated with HIV-Aids [79, 82, 83].	3
	Cinnamomum salicifolium (Nees) Kosterm.	H	Bulb; Siina (S)	Boiled and taken orally	Opportunistic infections associated with HIV-AIDS [82, 83].	2
	Cryptocarya myrtifolia Stapf.	SH	Stem bark; Camphor Tree (E); Igqebe (Z); Isithungwa (X); Kanferboom (A); Umgqebe (X); Umkhondweni (Z); Umncatyana (X); Umngqabe (Z); Umqqebe (X)	Not mentioned	"Isiphatsholo", STI associated with females [62, 64].	2
**Liliaceae**	*Albuca nelsonii* N.E Br	SH	Bulbs; Inqwebeba (Z/X)	Decoction taken orally	Gonorrhoea [39, 40].	2
**Lamiaceae**	*Clerodendrum glabrum* E.Mey.	SH	Stem bark; Mohlokohloko (S); Munkhathingwe (Tsh)	Dried, boiled in water and drunk	Oral ulcers associated with HIV-AIDS and various hepatitis infections [50, 57, 91].	3
	Leonotis leonurus (L.) R.Br.	SH	Whole plant; Bulderdagga (A); Cape Hemp (E); Dagga (A);	Not mentioned	Unidentified STIs and Hepatitis B [90].	1

			munyamunyane (Z); Imvovo (X); Lebake (S), Leonotis (E); Lion's Ear (E); Umfinafincane (X); Utshwala-bezinyoni Omncane (Z)			
	Mentha longifolia (L.) L.	H	Roots; Balderja (A); Horsemint (E); Kerena (S); Kruie (A); Pennyroyal (E); Rivier Kruie (A)	Not mentioned	Gonorrhoea, shingles associated with HIV-AIDS [9, 60, 92].	3
	Plectranthus ciliatus E.Mey.	H	Roots; Gespikkelde Muishondblaar (A); Lephele-phele (S); Speckled Spur-flower (E); Umsuthuza (Z)	Not mentioned	Opportunistic infections associated with HIV-AIDS [80, 83].	2
**Loganiaceae**	*Anthocleista grandifolia* Gilg	T	Stem bark and leaves; tobacco tree (E); Wildetabakboom (A); Mueneene (Tsh); Geludzu (X); Mophala (S)	Not mentioned	Unidentified STIs [95].	1
	Strychnos decussata (Pappe) Gilg	T	Stem bark; Cape Teak (E); Kaapsekiaathout (A); Mukangala (V); Umhlamahlahla (X); Umlahlankosi (Z)	Not mentioned	Various unidentified STIs [49].	1
	Strychnos madagascariensis Poir.	T	Roots and stem bark, Morutla (S), Mukwakwa (Tsh)	Ground onto powder and boiled, taken orally	Various unidentified l STIs, mouth ulcers associated with [19, 41, 57].	3

	Strychnos potatorum L.f.	T	Stem bark, Mukongovhoti (Tsh)	Soaked in cold water, used as mouth wash	Mouth ulcers associated with HIV-AIDS [57].	1
	Strychnos spinosa Lam.	T	Roots, Morapa, Mokwakwa (S)	Decoction taken orally	Various unidentified STIs [41].	1
**Loranthaceae**	*Erianthemum dregei* (Eckl. & Zeyh.) Tiegh.	SH	Root, Isidumbu (Z); Amaphakama (Z); Inevu emhlophe (Z); Inomfi (Z); Voëlent (A)	Not mentioned	Various unidentified STIs and internal sores [44].	1
**Malvaceae**	*Adansonia digitata* L.	T	Stem bark, Motsoo (S)	Not mentioned	Mouth ulcers and skin fungal infections associated with HIV-Aids [19].	1
	Grewia occidentalis L.	SH	Roots, Mopharatshwene (S)	Not mentioned	General STIs [68].	1
	Grewia flava DC.	H	Roots, Mothetlwa (S)	Decoction taken orally	Various unidentified STIs, chlamydia and diarrhoea arising from HIV-AIDS [19, 41, 59, 65].	4
	Grewia microthyrsa K.Schum. ex Burret	SH	Roots, Mupfuka (Tsh)	Not mentioned	Impotence in men [50].	1
	Grewia villosa Willd. var. *villosa*	SH	Roots; Mallow Raisin (E); Mupuna (Tsh); Murapfa (TSH); Tshirabva (Tsh)	Not mentioned	Vaginal discharge [76].	1
	Hibiscus vitifolius L.	SH	Roots, Muhwidzi (Tsh)	Not mentioned	Vaginal discharge [50].	1
	Hibiscus nigracaulis Baker f.	H	Roots; Mendonca (E)	Chewed	Impotence in men [53].	1
	Waltheria indica L.	H	Roots, Mokhutesela, Motayabannyana (S);	Boiled and taken orally	Various unidentified STIs [19, 41].	2

			Meidebossie (A); Delelemukula (Tsh)			
**Meliaceae**	*Ekeberkia capensis* Sparm.	T	Whole plant, Mmaba (S); Mmidibidi (S); Mountain ash (E), Mudouma (Tsh); Plum (E); Rooi-essenhout (A); Umathunzini-we-zintaba (Z); Umnyamathi (Z); Umnyamati (X)	Not mentioned	Various unidentified STIs [68, 75].)	2
	Melia azeadarach L.	T	Leaves; Mosara (S), Muserenga (Tsh)	Not mentioned	Gonorrhoea and shingles arising from HIV-AIDS [19, 60, 65].	3
	Trichilia dregeana Sond.	T	Leaves, Umkhuhlu (Z); Cape Mahogany (E); Forest Mahogany (E); Mmaba (S); Mutshikili (Tsh); Mutuhu (Tsh), Umkhuhlu (Z); Umathunzi (Z); Umhlakele (X)	Boiled and taken orally	Syphilis [44].	1
	Trichilia emetica Vahl.	T	Stem bark; Umkhuhlu (Z); Cape Mahogany (E); Forest Mahogany (E); Mmaba (S);	Decoction drunk	Impotence in men [53].	1
**Melianthaceae**	*Bersama lucens* (Hochst.) Szyszył.	T	Stem bark, Sindiyandiya (Z); Isindlandla (Z); Glossy bersama (E); Undlandla (X); Isindiyandiya (X);	Decoction taken orally	"Nta shete" and impotence [39, 40, 90, 91].	4

			Blinkblaarwitessenhout (A); Glossy white ash (E);			
**Menispermaceae**	*Cissampelos capensis* L.f.	SH	Roots; Davidjies (A); Davidjieswortel (A); Dawidjies (A); Dawidjieswortel (A); Fynblaarklimop (A)	Not mentioned	Syphilis [90, 93].	2
	Cissampelos torulosa E.Mey. ex Harv. & Sond.	T	Stem bark; Lukandululo (Tsh); Davidjies (A); Davidjieswortel (A); Ukhalimele (Z); Ukhalimele-omkhulu (Z), Umthombo (Z)	Ground onto powder and boiled, taken orally or gargled	Mouth ulcers associated with HIV-AIDS [57].	1
	Tinospora fragosa Verdoorn & Troupin	H	Leaves and stem; Marvel Creeper (E); Moses' Staff (E); Moses-se-kierie (A); Wonderplant (A); Wonderstok (A)	Boiled and taken orally	Various unidentified STIs [41].	1
**Mesembryanthemaceae**	*Carpobrotus edulis* (L.) N.E.Br.	H	Leaves, Tima mollo (S); Lutele (Tsh); Hottentot's Fig (E); Hottentotsvy (A); Ikhambilamabulawo (Z)	Dried, ground and immersed in water, used as mouth wash, or taken orally	Treat "tshofela", Herpes virus, mouth ulcers, shingles, and vaginal thrush associated with HIV-AIDS [19, 56, 57, 90, 91, 93, 96].	7
**Moraceae**	*Ficus abutilifolia* (Miq.) Miq.	T	Roots and stem bark, Mphaya (S); Mofa (S); Monokane (S); Tshikululu (Tsh); Ubambematsheni (Z); Large-leaved Rock Fig (E), Rock Wild Fig (E)	Boiled and taken orally	Various unidentified STIs [41].	1

	Ficus capensis Thunb.	T	Fruits; Muhuyu-ngala (TSH); Broom cluster fig (E)	Not mentioned	Various unidentified STIs [49].	1
	Ficus carica L.	T	Fruits, Muhuyu (Tsh); Common fig (E)	Latex mixed with water and used as mouth wash	Mouth ulcers associated with HIV-AIDS [57].	1
	Ficus sycomorous L.	T	Fruits; Muhuyu-lukuse (TSH); Sycomore fig (E)	Not mentioned	Various unidentified STIs (Fernandes et al., 2008)	1
	Morus alba L. var. *alba*	T	Whole plant; Moerbei (A); White Mulberry (E); Wit Moerbei (A)	Boiled and taken orally	Treats "divhu" [53].	1
**Musaceae**	*Ensete ventricosum* (Welw.) Cheesman	T	Roots, Banana (S); Banana palm (E); Wild Banana (E); Banana Tree (E); Motolô (S); Mulolo (Tsh), Piesangpalm (A)	Bioled and taken orally	Various unidentified STIs [41].	1
	Musa acuminata Colla	SH	Roots, Ubhanana (Z); Mobhanana (S)	Boiled and taken orally	Various unidentified STIs and internal sores [44].	1
**Myrothamnaceae**	*Myrothamnus flabellifolia* Welw.	SH	Whole plant, Boka (S); Feya (S); Ergboegoe (A); Bergbuchu (A); Resurrection plant (E); Umafavuke (ND); Uvukakwabafile (Z); Uvukwababa (Z)	Pounded and taken orally	Opportunistic infections associated with HIV-AIDS and Impotence [46, 65, 80, 81, 83].	5
**Myrtaceae**	*Psidium guajava* L.	T	Leaves, Mo-guava (S); common guava (E); yellow	Not mentioned	Various unidentified STIs, gonorrhoea [38, 50, 51, 89].	4

			guava (E), lemon guava (E)			
	Syzygium cordatum Hochst. ex Krauss	T	Leaves and stem bark, Umdoni (Z); Motlho (S); Motu (S); Mutu (Tsh); Umdoni (Z); Umjomi (X); Water berry (E); Water Wood (E); Waterbessie (A)	Boiled and taken orally	Gonorrhoea [38, 44, 89].	3
**Myrsinaceae**	*Rapanea melanophloeos* (L.) Mez	T	Stem bark; Swartbas (A); Swartbasboom (A); Tshididiri (Tsh); Tshikonwa (Tsh), Umaphipha (Z); Umaphiphakhubalo (Z), Uvukakwabafilikhubalo (Z)	Not mentioned	Various unidentified STIs [49].	1
**Ochnaceae**	*Brackenridgea zanguebarica* Oliv.	SH	Roots; Geellekkerbreek (A); Mutavhatsindi (Tsh)	Not mentioned	Various unidentified STIs [49]. (Fernandes et al., 2008)	1
**Olacaceae**	*Ximenia americana* L.	SH	Roots; Motshidi-mphiswane (S); Blue Sour Plum (E); Doringpruim (A); Suurpruim (A)	Not mentioned	Various unidentified STIs, mouth ulcers associated with HIV-AIDS and impotence [19, 82].	2
	Ximenia caffra Sond.	T	Roots, Motshidi-kgomo (S); Motshidi (S); Morotologa kgomo (S); Umgwenya (Z), Umthunduluka (Z), Sourplum (E); Wildesuurpruima (A)	Boiled and taken orally	Various unidentified STIs and opportunistic infections associated with HIV-AIDS and gonorrhoea [19, 41, 44, 49, 50, 65, 68-70, 76, 86].	11

**Oleaceae**	*Jasminum fluminense* Vell. subsp. *Fluminense*	SH	Roots; Lowveld Wild Jasmine (E); Maloyana (Z)	Boiled and taken orally	Various unidentified STIs [88].	1
**Orchidaceae**	*Anselia africana* Lindl.	H	Stem; Imfeyenkawu (Z); Leopard Orchid (E); Luiperdorgidee (A); Tiger Orchid (E); Tree Orchid (E)	chewed	Impotence in men [53].	1
**Papaveraceae**	*Argemone ochroleuca* Sweet.	H	Whole plant; Zavhazavha (Tsh); Bloudissel (A); Burweed (E); Geelpoppie (A); Mexican Poppy (E); Yellow Poppy (E)	Not mentioned	Gonorrhoea [60].	1
**Passifloraceae**	*Adenia gummifera* (Harv.) Harms	C	Roots; Phindaumshaye (Z); Impinda (Z); Impindamshaye (Z); Monkey Rope (E); Slangklimop (A); Snake-climber (E); Wild Grenadilla (E)	Boiled and taken orally	Opportunistic infections associated with HIV-AIDS and gonorrhoea [44, 53].	2
**Pedaliaceae**	*Dicerocarum eriocarpum* (Decne) Abels	H	Whole plant; Boot protectors (E); Devil thorn (E); Inkunzane (Z); Intekelane (Z) Stud thorn (E)	Boiled and the entire 1L IS taken orally	Syphilis [92].	1
	Harpagophytum procumbens (Burch.) DC. ex Meisn.	H	Fleshy roots; Lemata (S); Sengaparile (S); Devil's claw (E)	Boiled and taken orally	Chlamydia [59, 66].	2
**Phyllanthaceae**	*Antidesma venosum* E. Mey. ex Tul.	SH	Roots; Isibhangamlotha (Z); Isiqutwane (Z);	Boiled and drunk orally	Impotence in men [53].	1

			Kgôbê-tsabadishana (S); Modulane (S); Mufhala-khwali (Tsh); Tassel-berry (E); Tosselbessie (A); Umhlabahlungulu (Z); Umhlalanyoni (Z); Umtyongi (X)			
	Bridelia cathartica Bertol.	SH	Leaves and roots, Umkwawulangazi (Z); Blou-soetbessie (A); Blue Sweetberry (E)	Boiled and taken orally	Various unidentified STIs and internal sores [44].	1
	Bridelia micrantha (Hochst.) Baill.	SH	Stem bark; Isihlalamangcwibi (Z); Mitserie (A); Mitzeeri (E); Motsêrê (S); Munzere (Tsh); Mzeriehout (A); Umhlahlahlungulu (X)	Boiled and taken orally	Various unidentified STIs and internal sores [50].	1
	Bridelia mollis Hutch.	SH	Stem bark; Mokamanawa (S); Mokokonala (S); Mukumba-kumbane (Tsh); Velvet Bridelia (E); Velvet Sweet-berry (E)	Not mentioned	Various unidentified STIs [84].	1
	Pseudolachnostylis maprouneifolia Pax var. *dekindtii* (Pax) Radcl.-Sm.	T	Stem bark; Hairy Kudu-berry (E); Harige Koedoebessie (A)	Not mentioned	Various unidentified STIs [50, 76].	2
**Picrodendraceae**	*Androstachys johnsonnii* Prain.	T	Roots; Lebombo ironwood (E)	Boiled and taken orally	Impotence in men [53].	1
**Piperaceae**	*Piper capense* L.f.	SH	Stem bark, Mulilwe (Tsh); Ihlolane (Z);	Dried bark is ground and	Various unidentified STIs, vaginal discharge and	3

			Uluphokwane (Z), Uluphokwana (Z); Sterkpeper (A); Stertpeper (A); Wild Pepper (E),	boiled, taken orally	opportunistic infections associated with HIV-AIDS [49, 50, 57].	
**Pittosporaceae**	*Pittosporum viridiflorum* Sims	T	Roots and stem bark; White Cape Beech (E); Witboekenhout (A); Umphushane (Z); Kgalagangwe (S); Mutanzwakhamelo (Tsh); Umgqwengqwe (X)	Powdered plant material is taken with beer.	Impotence in men [95].	1
**Polygalaceae**	*Persicaria decipiens* (R.Br.) K.L.Wilson	H	Whole plant, Mohwehwe (S)	Not mentioned	Gonorrhoea [93].	1
	Polygala fruticosa P.J.Bergius	H	Whole plant; Featherdusters (E); Heart-leaved Polygala (E); Ithethe (Z); Shrubby Polygala (E)	Not mentioned	Gonorrhoea [38, 90, 91].	3
	Securidaca longipedunculata Fresen.	SH	Roots, Mphesu (S); Mmaba (S); Mupesu (Tsh)	Powdered and take with mageu orally for aphrodisiac use and decoction taken orally for chlamydia	Used to treat impotence in men treats chlamydia, gonorrhoea and various unidentified STIs [19, 46, 49, 50, 56, 59, 63, 76, 81, 91].	10
**Portulacaceae**	*Portulaca quadrifolia* L.	H	Whole plant; Amalenjane(Z); Ushisizwe (Z); Usompungane	Not mentioned	Pubic lice [61].	1

**Proteaceae**	*Faurea saligna* Harv.	T	Stem bark, Mohlakô (S); Mongêna (S); Mutango (Tsh); Rooi-boekenhout (A); Swartboekenhout (A); Transvaal Beech (E); Transvaal Beechwood (E); Isefu (Z); Isisefo (Z)	Dried, powdered and soaked in warm water and then used as douched for vaginal ulcers	Vaginal ulcers [57, 86].	2
	Protea caffra Meisn.	T	Seeds, Gewone suikerbos (A); Highveld Protea (E), Natal Sugarbush (E); Indlunge (X); Isiqwane (X); Isadlunge (X); Isiqalaba (Z); Mogalagala (S); Segwapi (S); Tshididiri (Tsh); Uhlinkihane (Z);	Not mentioned	Chlamydia [80, 83].	2
**Punicaceae**	*Punica granatum* L.	SH	Roots, Mokgarenate (S)	Boiled and taken orally	Diarrhoea and oral thrush associated with HIV-AIDS [19, 43].	2
**Ranunculaceae**	*Ranunculus multifidus* Forssk.	H	Whole plant, Uxaphozi (Z); Ishashakazane (Z); Isijojokazana (Z); Botterblom (A); Kankerblare (A); Buttercup flower (E)	Decoction used as enema	Genital warts and gonorrhoea [44, 72].	2
**Rhamnaceae**	*Helinus intergrifolius* (Lam.) Kuntze	T	Roots, Morakane (S)	Boiled and taken orally	Various unidentified STIs [41].	1
	Ziziphus mucronata Willd.	T	Roots, Mokgalo (S); Mukhalu (Tsh); Mutshetshete (Tsh); Skynblaar-wag-'n-bietjie	Boiled and taken orally	Various unidentified STIs, impotence, gonorrhoea, diarrhoea, and fungal infections associated with	8

			(A); Umhlahlankosi (Z); Umlahlankosi (Z); Umpafa (ND)		HIV-AIDS [19, 41, 45, 53, 65, 75, 80, 83].	
	Berchemia discolor (Klotzsch) Hemsl.	T	Stem bark and leaves; Motsintsila (S); Mountain Date (E); Muhukhuma (Tsh); Munie (Tsh); Nmumu (Z); Wild Almond (E); Wild Date (E); Wilde-dadel (A)	Not mentioned	Vaginal discharge [76].	1
**Rosaceae**	*Parinari curatellifolia* Planch. ex Benth.	T	Stem bark, Muvhula (TSH); Mobola Plum (E); Muchakata (ND); Muvhula (Tsh); Sand Apple (E); Ubulawu (Z); Umnkuna (ND)	Not mentioned	Various unidentified STIs [50].	1
	Prunus africana (Hook.f.) Klakman	T	Roots, Inkokhokho (Z); Inyazangoma (X); Inyazangomelimnyama (Z); Itywina-elikhul (X); Mogohloro (S); Mulala-maanga (Tsh)	Not mentioned	Various unidentified STIs and opportunistic infections associated with HIV-AIDS [9, 75].	2
	Prunus persica (L.) Batsch	T	Roots; Moperekisi (S); Pygeum (E)	Powdered and taken with mageu/porridge	Impotence in men [46, 53, 65, 78, 81, 71].	6
**Rubiaceae**	*Cepalanthus natalensis* Oliv.	SH	Roots; Far Tree (E); Murondo (Tsh); Quinine Berry (E); Strawberry-bush (E); Witpruim (A)	Decoction taken orally	Impotence in men [53].	1

	Lagyniasis dryadum (S.Moore) Robyns	SH	Roots; Mmilo o monnyane (S)	Decoction taken orally	Impotence in men [53].	1
	Coddia rudis (E.Mey. ex Harv.)	SH	Whole plant; Kleinbeenappel (A); Lesser Xeromphis (E); Mukwakwa (Tsh); Small Bone-apple (E); Tshinapanapana (Tsh); Umdondwani (Z)	Not mentioned	Impotence in men [64].	1
	Richardia brasiliensis Gomes	H	Whole plant; Mulegere (Tsh); Molekere (S)	Dried, burnt and mixed with animal fat, applied directly to infected mouth and vaginal ulcers	Mouth and vaginal ulcers [57].	1
	Toddalia asiatica L.	C	Roots and leaves; Climbing Orange (E); Cockspur Orange (E); Rank-lemoentjie (A)	Decoction drunk	Unidentified STIs [53].	1
	Vangueria infausta Burch.	T	Roots and stem bark; Mmilo (S); Ntswila (S); Mizwilu (Tsh); Muzwilu (Tsh); Grootmispel (A); Velvet Wild-medlar (E); Wildemispel (A)	Chopped in small pieces and boiled, taken orally	Stem bark is used to treat diarrhoea associated with HIV-AIDS, Roots are used for impotence in men [42, 50, 57, 62, 64].	5
	Vangueria pygmaea Schltr.	T	Stem bark and leaves; Dwarf Crowned-medlar (E); Goubos (A); Gousiekte	Not mentioned	Unidentified STIs [85].	1

			Bush (E); Grysappel (A); Hairy Gousiektebossie (E); Umkukuzela (Z)			
**Rutaceae**	*Agathosma apiculata* E.Mey. ex Bartl. & H.L. Wendl.	SH	Leaves; Knoffel Buchu (E); Knoffelboegoe (A)	Boiled and taken orally	Shingles, mouth, and genital ulcers associated with HIV-AIDS [9].	1
	Citrus limon (L.) Osbeck	SH	Roots; Tshikavhavhe (Tsh)	Boiled and taken orally	General STIs [50].	1
	Zanthozylum capense (Thunb.) Harv.	T	Roots and leaves, Senokomaropa (S), Umlungumabele (X)	Infusions taken orally	Syphilis and opportunistic infections associated with HIV-AIDS [40-42, 65, 80, 83].	6
	Zanthozylum davyi (I.Verd.) P.G.Waterman	T	Roots; Munungu (Tsh); Senokomaropa (S); Tshamavhudzi (Tsh); Umlungamabele (X); Umlungumabele (X); Umnungumabele (Z)	Not mentioned	Unidentified STIs and impotence [49, 50].	2
	Zanthozylum humile (E.A.Bruce) P.G.Waterman	SH	Roots; Monokonokwane (S); Hairy knobwood (E); Harige perdepram (A)	Powdered and taken with mageu or soft porridge	Opportunistic infections associated with HIV-AIDS and impotence [46, 80-83].	4
**Salicaceae**	*Oncoba spinosa* Forssk subsp. *Spinosa*	SH	Roots; Snuff-box Tree (E); Tonga (A)	Decoction taken orally	Impotence in men [53].	1
**Santalaceae**	*Osyris lanceolata* Hochst. & Steud.	SH	Roots, Mphere (S); Serogabaloi; Mpeta (Tsh); ladbas (A), Ingondotha-mpete (Z), Intekaza (X)	Boiled and taken orally	Various unidentified STIs, opportunistic infections associated with HIV-AIDS (oral thrush) and impotence [46, 50, 57, 59, 68-70].	6

	Pappea capensis Eckl. & Zeyh.	T	Leaves; Murodolo (Tsh); Mongatane (S); Morobadiepe (S) Umvuma (Z), Uvumebomvu (Z), Uzagogwane (Z), Wild Cherry (E), Wild Plum (E), Wilde-amandel (A), Xikwakwaxu (TS)	Ground and immersed in water	Various unidentified STIs, Vaginal ulcers associated with HIV-AIDS, chlamydia and as an aphrodisiac for men [50, 53, 57, 59, 68-70].	7
	Viscum capense L.f.	SH	Stems; Voëlent (A); Vogelstront (A)	Decoction drunk as tea	Mouth ulcers associated with HIV-AIDS [43].	1
**Sapindaceae**	*Dodonaea viscosa* Jacq var. angustifolia (L.f) Benth.	T	Roots; Mofentshe (S); Muthathavhanna (Tsh); mutata-vhana (Tsh); Sand olive (E); Sandolien, ysterbos (A)	Fresh leaves are crushed and macerated in water, drunk	Opportunistic infections associated with HIV-AIDS, Oral thrush [53, 57, 65, 80, 82, 83].	6
**Sapotaceae**	*Englerophytum magalismontanum* (Sond.) T.D.Penn.	SH	Roots; Amanumbela (Z); Milkplum (E); Mohlatswa (S), Munombelo (Tsh), Stamvrug (A)	Boiled and taken orally	Unidentified STI s and impotence [65].	1
	Mimusops caffra E.Mey. ex A.DC.	T	Roots; Amasethole (Z); Coastal Red Milkwood (E); Rooimelkhout (A)	Boiled and taken orally	Gonorrhoea [44].	1
	Mimusops zeyheri Sond.	T	Roots, Monupudu (S); Mububulu (Tsh); Mutaladzi (Tsh); Red Milkwood (E); Rooimelkhout (A); Umbumbulu (Z)	Boiled and taken orally	Syphilis [19, 65].	2
**Solanaceae**	*Capsicum annum* L. var *glabriusculum*	SH	Roots; fruits and leaves; Mutunbutumbula (Tsh)	Boiled and taken orally	Treat "divhu" [53].	1

	(Dunal.) Heiser & Pickersgill					
	Capsicum frutescens L.	SH	Roots, fruit, and leaves; African Chilli (E); African Pepper (E); Natal Chilli (E); Red Pepper (E)	Not mentioned	Treat "divhu" [53].	1
	Datura stramonium L.	SH	Leaves; Zavhazavha (Tsh); Jimsonweed (E), Thornapple (E).	Not mentioned	Various unidentified STIs, gonorrhoea, impotence [50, 71, 90, 91].	4
	Nicotiana glauca Graham	SH	Roots; Tobacco tree (E); Wild tobacco (E)	Crushed and boiled	Gonorrhoea and syphilis [51].	1
	Solanum aculeastrum Dunal subsp. *aculeastrum*	H	Fruits; Goat Bitter Apple (E); Intuma (Z); Umthuma (Z); Murulwa (Tsh); Poison-apple (E); Shulwa (Tsh)	Boiled and taken orally	General STIs [49, 61].	2
	Solanum campylacanthum Hochst. ex. A.Rich. subsp. *panduriforme*	H	Roots; Bitter Apple (E); Bitterappel (A); Intuma (Z); Intuma-omncane (Z); Moralane (S); Morolwana (S); Poison Apple (E);	Boiled and taken orally	Gonorrhoea and "divhu" [53].	1
	Solanum catombelense Peyr.	SH	Roots; Morola o monnyane (S)	Decoction taken orally	Chlamydia [59].	1
	Solanum elaeagnifolium Cav.	SH	Roots or stems; Morola (S)	Bioled and taken orally. In case of genital sores, its applied topically mixed with Vaseline	Gonorrhoea, chlamydia, syphilis, genital warts, and opportunistic infections associated with HIV-AIDS [51, 52].	2

	Solanum linnaeanum Hepper & Jaeger	SH	Roots; Bitter Apple (E)	Boiled and taken orally	Gonorrhoea [93].	1
	Solanum nigrum L.	H	Fruit; Muxe (Tsh)	Green berries are pounded and mixed with water, taken orally	Opportunistic infections associated with HIV-AIDS [57].	1
	Solanum mauritianum Scop.	SH	Roots; Bugweed (E); Morola (S)	Boiled and taken orally	Gonorrhoea, chlamydia, syphilis, "makgoma" and opportunistic infections associated with HIV-AIDS [51].	1
	Solanum panduriforme E. Mey.	H	Fruits; Thola e serolwane (S); Morolwana (S); Ndhulwani (Tsh); Intumemncane (Z)	Chopped and macerated in water and taken orally	Gonorhea and opportunistic infections associated with HIV-AIDS [45, 49, 57, 62, 64, 65, 71, 73, 79, 83].	10
	Withania somnifera (L.) Dunal.	H	Roots; Mosalamaropeng (S); Bofepha (S); Musalamarumbini (Tsh); Ubuvimbha (Z)	Boiled and taken orally	"Divhu", syphilis and mouth ulcers associated with HIV-AIDS [53, 57, 91, 92].	4
**Strelitziaceae**	*Strelitzea reginae* Ait	SH	Leaves; Bird-of-paradise Flower (E); Crane Flower (E); Geelpiesang (A); Piesangboom (A); Voëls (A), Wild Banana (E)	Boiled and drunk	Various unidentified STIs [38, 89].	2
**Thymelaceae**	*Lasiosiphon kraussianus* (Meisn.) Burtt Davy var. *kraussianus*	H	Whole plant; Inhlashane (Z); Isidikili (Z); Lesser Yellowhead (E); Thopa S) Umfukuzane (Z); Umsilawengwe (Z)	Not mentioned	Impotence [61].	1

**Typhacaceae**	*Typha capensis* (Rohrb.) N.E.Br.	H	Roots; Bulrush (E); Cat's Tail (E); Cossack Asparagus (E); Ibhuma (Z); Ingcongolo (X); Matjiesgoed (A); Motsitla (S)	Decoction is drunk three times a day	Various unidentified STIS, impotence [38, 61, 89, 90, 91].	5
**Ulmaceae**	*Celtis africana* Burm.f.	T	Roots; Modutu (S); Mogatakgomo (S); Mothibadifate (S); Mpopana (Tsh); Mumvumvu (Tsh); Ndwandwazane (Z); Umvumvu (Z); Umvumvu (X), Waterboom (A); White Stinkwood (E)	Boiled and taken orally	Impotence in men [73].	1
**Utricaceae**	*Obetia tenax* (N.E.Br.) Friis	T	Stem bark; Dyambila (Tsh); Gukhunya (Tsh); Imbati (Z); Impongozembe (Z); Lebati (S); Mountain Nettle (E); Muendanathavha (Tsh); Uluza (X)	Not mentioned	General STIs [49].	1
	Urtica dioica L.	H	Stem and leaves; Bobatsi (S); Brandnekel (A); Common Nettle (E); Great Nettle (E); Stinging Nettle (E); Swedish Hemp (E); Umbabazane (X)	Boiled and taken orally	Opportunistic infections associated with HIV-AIDS [9].	1
**Verbenaceae**	*Clerodendron glabrum* var *glabrum*	T	Roots and stem bark; Mutsho (S)	Boiled and taken orally	Oral candidiasis [57, 58].	2

	Lantana camara L.	SH	Leaves and twigs; Semela nageng (S); Mogwagwaila (S)	Crushed and boiled, taken orally	Chlamydia, gonorrhoea, syphilis, and opportunistic infections associated with HIV-AIDS [51].	1
	Lippia javanica	H	Whole plant; Bokhukhwane (S); Fever Tea (E); Inzinziniba (X); Koorsbossie (A); Koorsteebossie (A); Umsuzwane (Z); Umswazi (Z)	Decoction of a plant is used to wash infected area	Pubic lice [61, 91].	2
	Phyla nodiflora (L.) Greene	H	Roots; Tshishengelaphofu (Tsh)	Chewed and spit out	Oral thrush associated with HIV-AIDS [54].	1
**Violaceae**	*Rinorea angustifolia* (Thouars) Baill.	SH	Roots; Mafambaborile (Tsh); Narrow-leaved Violet-bush (E)	Boiled and taken orally	General opportunistic infections associated with HIV-AIDS [57].	1
**Vitaceae**	*Cissus quadrangularis* L. var. *quadrangularis*	H	Stem bark; Mohlabadipoo (S); Cactus vine (E); Umnhlonhlwanyane (Z)	Boiled and taken orally	Various unidentified STIs and skin infections associated with HIV-AIDS [19, 66, 89].	3
	Rhoicissus tridendata (L.f.) Wild & R.B.Drumm.	SH	Roots; Terbe ya naga (S)	Not mentioned	Various unidentified STIs and against impotence [53, 76, 87, 88].	4
**Zingiberaceae**	*Siphonochilus aethiopicus* (Schweinf.) B.L.Burtt	H	Bulb; Indungulu (Z); Isiphephetho (Z); Wild Ginger (E)	Not mentioned	Various unidentified STIs [75].	1
**Zygophyllaceaea**	*Tribulus terrestris* L.	SH	Whole plant, Mosehlo (S), Tsetwana (TSH); Puncture Vine (E)	Not mentioned	Roots are used to treat chlamydia or "go khutlega" while whole plant is used to treat syphilis [65, 79, 83].	3

Key: A, Afrikaans; E, English; Tsh, TshiVenda; Z, IsiZulu; X, IsiXhosa; S, Sepedi; Tsh, TshiVenda, ND, Ndebele; Ts, Tswana
